# Identifying important parameters in the inflammatory process with a mathematical model of immune cell influx and macrophage polarization

**DOI:** 10.1371/journal.pcbi.1007172

**Published:** 2019-07-31

**Authors:** Marcella Torres, Jing Wang, Paul J. Yannie, Shobha Ghosh, Rebecca A. Segal, Angela M. Reynolds

**Affiliations:** 1 Department of Mathematics and Applied Mathematics, Virginia Commonwealth University, Richmond, Virginia, United States of America; 2 Department of Internal Medicine, Virginia Commonwealth University, Richmond, Virginia, United States of America; 3 Hunter Holmes McGuire VA Medical Center, Richmond, Virginia, United States of America; 4 Victoria Johnson Center for Lung Disease Research, Virginia Commonwealth University, Richmond, Virginia, United States of America; University of Utah, UNITED STATES

## Abstract

In an inflammatory setting, macrophages can be polarized to an inflammatory M1 phenotype or to an anti-inflammatory M2 phenotype, as well as existing on a spectrum between these two extremes. Dysfunction of this phenotypic switch can result in a population imbalance that leads to chronic wounds or disease due to unresolved inflammation. Therapeutic interventions that target macrophages have therefore been proposed and implemented in diseases that feature chronic inflammation such as diabetes mellitus and atherosclerosis. We have developed a model for the sequential influx of immune cells in the peritoneal cavity in response to a bacterial stimulus that includes macrophage polarization, with the simplifying assumption that macrophages can be classified as M1 or M2. With this model, we were able to reproduce the expected timing of sequential influx of immune cells and mediators in a general inflammatory setting. We then fit this model to *in vivo* experimental data obtained from a mouse peritonitis model of inflammation, which is widely used to evaluate endogenous processes in response to an inflammatory stimulus. Model robustness is explored with local structural and practical identifiability of the proposed model *a posteriori*. Additionally, we perform sensitivity analysis that identifies the population of apoptotic neutrophils as a key driver of the inflammatory process. Finally, we simulate a selection of proposed therapies including points of intervention in the case of delayed neutrophil apoptosis, which our model predicts will result in a sustained inflammatory response. Our model can therefore provide hypothesis testing for therapeutic interventions that target macrophage phenotype and predict outcomes to be validated by subsequent experimentation.

## Author summary

Using experimental data and mathematical analysis, we develop a model for the inflammatory response that includes macrophage polarization between M1 and M2 phenotypes. Dysfunction of this phenotypic switch can disrupt the timely influx and egress of immune cells during the healing process and lead to chronic wounds or disease. The modulation of macrophage population has been suggested as a strategy to dampen inflammation in diseases that feature chronic inflammation, such as diabetes and atherosclerosis. It is therefore important that we learn more about which components of the system drive the population level switch in phenotype. Our model is able to reproduce the expected timing of sequential influx of neutrophils and macrophages in response to an inflammatory stimulus. Model parameters were estimated with weighted least squares fitting to *in vivo* experimental data from a mouse model of peritonitis while considering identifiability of parameter sets. We perform sensitivity analysis that identifies primary drivers of the system, and predict the effects of variations in these key parameters on immune cell populations.

## Introduction

Macrophages play an essential role in both the progression and the resolution of inflammation. These contradictory roles may be explained by the idea of a spectrum of macrophage phenotypes, ranging from the inflammatory M1 phenotype to the anti-inflammatory M2 phenotype at either extreme, with diverse subpopulations of macrophages in between [1–4]. Another possible explanation is that macrophages exhibit typically M1 or M2 type functions to varying degrees at various points in time, or there are portions of each type present at each of the different phases of inflammation [5]. While this duality of purpose is not fully understood, it is known that an imbalance between pro- and anti-inflammatory macrophage activities has been linked to disordered healing and implicated in many inflammatory diseases. For example, overpopulation of M1 macrophages can induce tissue injury [1], and the accumulation of M1s in adipose tissue which secrete pro-inflammatory cytokines can lead to insulin resistance, diabetes, and atherosclerosis [6, 7]. Even M2 macrophages, which are thought of as resolving inflammation, can cause disorders such as allergies, asthma, fibrosis, and excessive scarring when present in large numbers [4]. There is also an increased association of M2 polarized macrophages with solid tumor formation [8, 9].

All macrophages begin life as monocytes circulating in the bloodstream and, upon settling into tissues and organs in the body, will adapt to their local environment. At an inflamed site, monocytes are triggered to differentiate into macrophages in response to stimuli such as chemokines and cytokines in the environment, phagocytosis of apoptotic cells or debris, or the presence of pathogen [1, 4, 7, 10, 11]. These first invading macrophages primarily activate to a more M1 phenotype but, under normal conditions, M2 macrophages producing anti-inflammatory cytokines will eventually dominate, suppressing the inflammatory and Th1 adaptive immune response, while promoting a Th2 response [4]. In response to infection or presence of pathogens, neutrophils are the first immune cell to appear to facilitate removal. Subsequent macrophage infiltration is essential for the removal of apoptotic neutrophils and continued secretion of cytokines to further limit the effects of the invading pathogens [12].

This timely recruitment and egress of immune cells is central to the mounting of an appropriate immune response that resolves to restore tissue homeostasis. Dysfunction or disruption of this response is the cause of essentially all chronic inflammatory diseases. Appropriate switching of phenotype of the overall macrophage population from initial M1 to M2 phenotype is critical for a balanced response. Knowledge of which subpopulations of macrophages to modulate is therefore necessary for the development of interventions that can aid in the resolution of inflammation.

Mathematical modeling has been extensively applied to the problem of inflammation in a variety of contexts such as wound healing [13–24] and atherosclerosis [25–32]. Deterministic ordinary differential equations (ODEs) in particular have been used when the primary interest is capturing time course and/or qualitative behavior at the cellular level. Reynolds et al. in 2006 [14] modeled the innate immune response to pathogen including activated phagocytes, level of pathogen, tissue damage, and anti-inflammatory mediators and this model was modified to apply to a local wound with the inclusion of fibroblast activity and the effect of tissue oxygen levels in Menke et al. [17]. The work was further extended by Segal et al. in 2012 [20], adding collagen accumulation as a means of tracking the healing progress. Cooper et al. [23] next tracked macrophages and neutrophils specifically rather than a single variable representing immune response. Phagocytosis of apoptotic neutrophils was considered a key driver of the resolution of inflammation in models developed by Dunster et al. [22]. In a study analyzing macrophage polarization following myocardial infarction, Wang et al. [21] tracked both M1 and M2 macrophages as well as pro- and anti-inflammatory mediators. Recent work by Lee et al. [33] models M1 and M2 macrophage response to respiratory viral infection along with epithelial cells, cytokines, and enzymes.

In this manuscript, we draw on the work done in these previous models to develop a new computational model of inflammation that seeks, in part, to explain the relationship between macrophage polarization and neutrophils. To our knowledge, our model is the first to include both inflammatory M1s and resolving M2s that is fit to *in vivo* experimental data.

We first use ODEs to develop a computational model of the sequential influx of immune cells in response to an external trigger to permit a system-level analysis of the processes. We then parametrize the model by fitting to cell count data for neutrophils, M1 macrophages, and M2 macrophages obtained from a mouse model of peritonitis, a well-accepted model to assess inflammatory responses *in vivo* that is also widely used to evaluate the efficacy of targeted anti-inflammatory interventions. This step entails finding a subset of identifiable parameters to estimate and fixing those that were unidentifiable, a process that has many approaches across a wide application area [34–39]. Once a final parameter set is estimated, we conduct a local sensitivity analysis of the fitted model in order to gain an understanding of the primary controls of the system. The results support the dependence of macrophage polarization on neutrophils that has been hypothesized in the literature [1, 3, 40–42]. Finally, we use the model to test several macrophage-targeted treatment scenarios that are hypothesized to dampen inflammation. The resulting predictions could have implications in the development of treatment strategies for chronic inflammation.

## Materials and methods

### Ethics statement

The use of animals for this study was approved by VCU IACUC and the approved protocol number is AM10346 with an approval date of 4-14-18 and this approval will expire on 3-13-2021. Isofluorane inhalation was used for euthanasia.

### Experimental details

**Fig 1:**
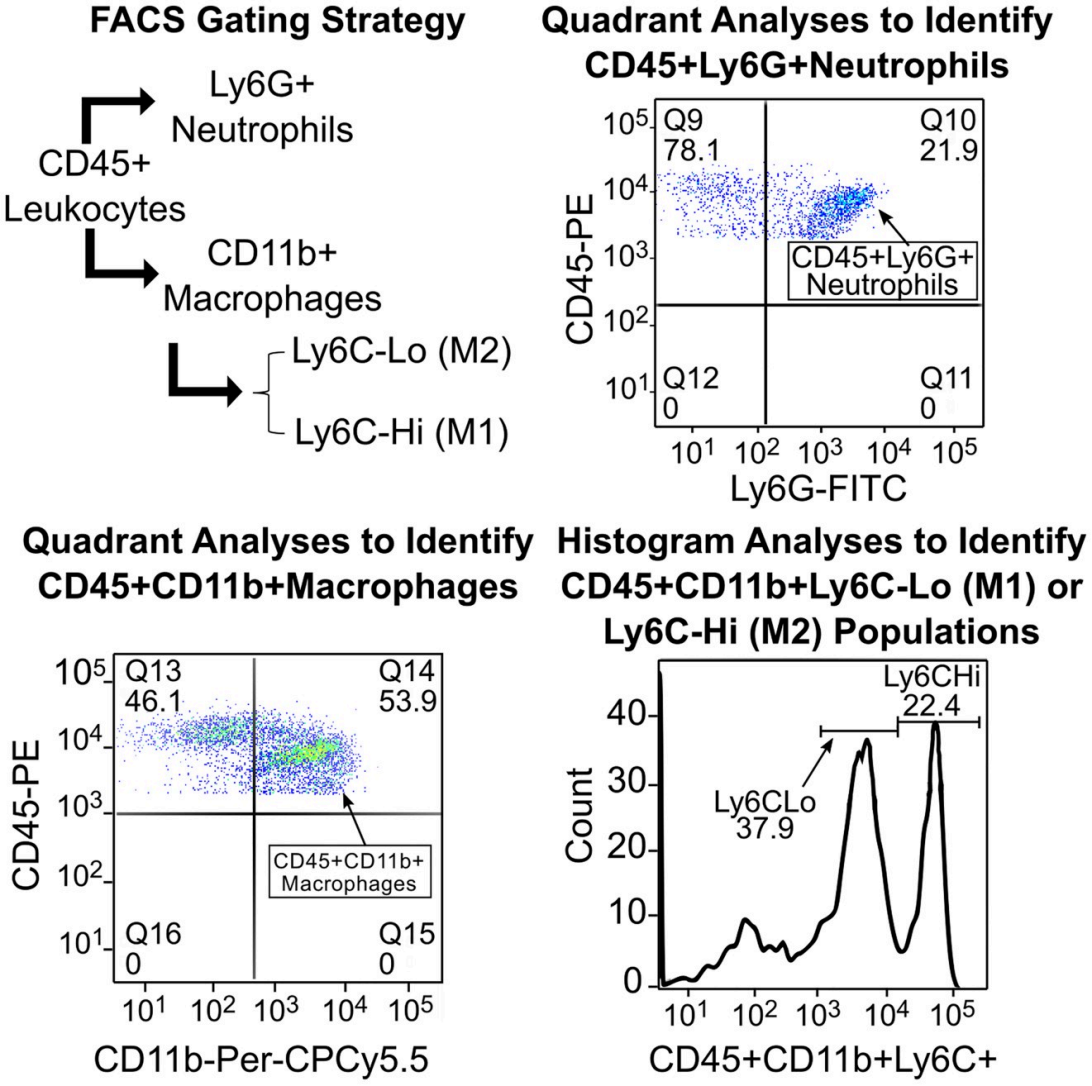
Experimental details. Gating strategy and representative dot plots and histograms used to identify individual cell populations.

Induction of peritonitis by intraperitoneal injection of thioglycollate broth, which will facilitate the rapid growth of bacteria in the peritoneal cavity, is a well-suited platform to monitor the influx of immune cells and also permits easy characterization of the infiltrating cells in a time dependent manner. Peritoneal exudates were harvested from mice at 10 different time-points over 7 days after a single intraperitoneal injection of 3% thioglycollate broth. The peritoneal cavity was flushed with serum free RPMI medium. The cells were collected by brief centrifugation, re-suspended, and then stained with fluorescently conjugated antibodies to CD45, CD11b, Ly6G (Gr-1), F4/80 and Ly6C and analyzed by flow cytometry to determine the distribution of neutrophils, macrophages and Ly6CHi (M1) or Ly6CLo (M2) polarization [43]. While all leukocytes are CD45+, neutrophils and macrophages can be distinguished by the presence of specific markers, namely Ly6G or Gr1 and CD11b or F4/80 on neutrophils and macrophages, respectively. The macrophages in the peritoneal exudates can further be differentiated into resident (CD11bHi and F4/80Hi), inflammatory M1 (CD11b+Ly6CHi) and anti-inflammatory M2 (CD11b+Ly6CLo) phenotypes. The gating strategy and representative dot plots and histograms used to identify individual cell populations are shown in Fig 1. Flow cytometry data was analyzed using the FlowJo software and percent distribution of individual cell type determined as described earlier [43]. The data collected from these experiments is used to calibrate the model parameters (see Supporting Information).

### Model development

**Fig 2:**
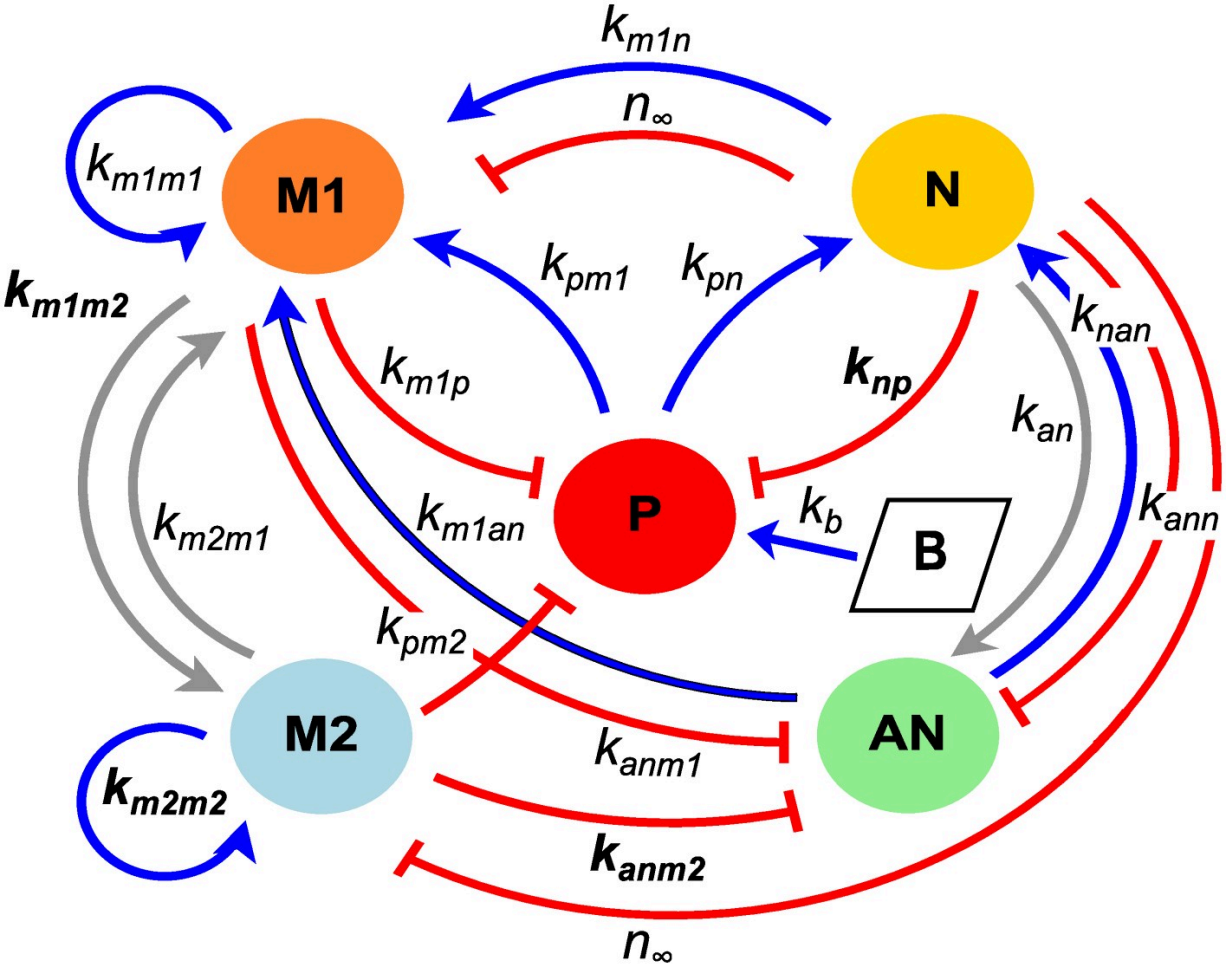
Model schematic. Model schematic for the inflammatory response with variables defined in the equations. Arrows represent up-regulation and bars represent destruction or inhibition. Parameters in the schematic that are included in the final subset of identifiable parameters appear in bold; additional non-interaction parameters that do not appear in the schematic are given with the full subset in Table 4.

The model developed in this manuscript tracks the signaling and resulting immune response within the peritoneal cavity. We do not explicitly model the blood component and all variables represent local levels. To create this model, previous models of immune response to a wound [14, 20, 23] have been adapted to include polarization of macrophages between phenotypes M1 and M2, transition of neutrophils to the apoptotic state, and the injection of nutrient broth to induce growth of pathogen and stimulate immune response. System variables include cell populations given by *M*1 (M1 macrophages), *M*2 (M2 macrophages), *N* (neutrophils), and *AN* (apoptotic neutrophils) as well as *P* (pathogen) and *B* (inflammatory stimulus). We track the total cells for each population with units of 10^7^ cells. Model parameters for rates of activation, transition, decay, and interactions are specified in Table 1. Units for many of the model parameters are given in terms of their associated variable, since they are representative of immune functions such as cell signaling and mediators for which units cannot be determined. The model is summarized in Fig 2 and described by Eqs 1–6.

**Table 1:** Description of parameters and estimates for the full model.

Parameter	Description	Initial Estimate	Bounds
*P*_0_	initial concentration of pathogen	set equal to *p*_∞_	(fixed)
*B*_0_	initial concentration of nutrient broth	1 × 10^3^/cm^3^	(fixed)
*k*_*b*_	destruction rate of B by P	10/day	(fixed)
*p*_∞_	pathogen carrying capacity	3 × 10^−3^/cm^3^	(1 × 10^−6^,1)
*k*_*pg*_	growth rate of pathogen	35/day	(10, 35)
*k*_*pn*_	destruction rate of pathogen by N	0.295/*N*-units/day	(0.11, 5)
*k*_*pm*_	destruction rate of pathogen by macrophages	6.11/*M*-units/day	(1, 10)
*s*_*mr*_	source of resting monocytes	21.440/*M*-units/day	(8, 100)
*μ*_*mr*_	decay of resting monocytes	5.156/day	(5,80)
*k*_*m*1*p*_	activation rate of M1 by P	1.00/*M*-units/day	(1, 5)
*k*_*m*1*n*_	activation rate of M1 by N byproducts	0.025/*M*-units/day	(0.01, 5)
*k*_*m*1*an*_	activation rate of M1 by AN	0.997/*M*-units/day	(1 × 10^3^, 5)
*k*_*m*1*m*1_	activation rate of M1 by M1s and their cytokines	0.001/*M*-units/day	(1 × 10^4^, 5)
*k*_*m*2*m*1_	transition rate of M2 to M1	0.117/*M*-units/day	(0.01, 1)
*μ*_*m*1_*	decay of M1 macrophages	6.956/day	(1, 20)
*k*_*m*1*m*2_*	transition rate of M1 to M2	8.281/*M*-units/day	(0.1, 100)
*k*_*m*2*m*2_*	activation rate of M2 by M2s and their cytokines	1.624/*M*-units/day	(1 × 10^3^, 5)
*k*_*c*_	concentration of background anti-inflammatory	0.0125/*M*-units/day	(fixed [14])
*μ*_*m*2_	decay of M2 macrophages	8.271/day	(1, 20)
*s*_nr_*	source of resting N	15.889/*N*-units/day	(10, 100)
*u*_*nr*_	decay of resting N	3.978/day	(1, 10)
*k*_np_*	activation rate of N by P	3.703/*N*-units/day	(1 × 10^3^, 50)
*k*_*nan*_	activation rate of N by AN	0.607/*N*-units/day	(0.0001, 5)
*n*_∞_	level of *N* for 50% inhibition of *M* activity	0.156/*N*-units	(0.01, 5)
*k*_*an*_	transition rate of N to AN	7.108/*N*-units/day	(1, 30)
*k*_*ann*_	destruction rate of AN by N	0.001/*N*-units/day	(1 × 10^6^, 0.01)
*k*_*anm*1_	destruction rate of AN by M1	2.898/*N*-units/day	(0.1, 1 × 10^3^)
*k*_*anm*2_*	destruction rate of AN by M1	87.080/*N*-units/day	(5, 1 × 10^3^)
*μ*_*an*_	secondary necrosis of AN	1.309/day	(1, 15)

The value for *k*_*c*_ was set to the value for *s*_*c*_, source of anti-inflammatory mediator, in Reynolds et al. [14]. To set bounds for parameter fitting, Latin Hypercube Sampling was used to find parameter sets that resulted in a physiologically reasonable range of responses. This process is described in detail in Cooper et al. [23]. Parameters included in the final subset of identifiable parameters, with all other parameters fixed, are marked with *, with final estimates given in Table 4.

Macrophages:

$$\begin{array}{c} \frac{dM1}{dt}=\overset{\text{activation/influx}\;\text{rate}}{\overset{︷}{\frac{s_{mr}R_{M1}\left(P,N,M1,AN\right)}{\mu_{mr}+R_{M1}\left(P,N,M1,AN\right)+R_{M2}\left(M2\right)}}}-\overset{\text{switch}\;\text{to}\;\text{M2}\;\text{from}\;\text{M1}\;\text{per}\;\text{phagocytized}\;\text{AN}}{\overset{︷}{k_{m1m2}k_{anm1}ANf_{i}\left(M1,N\right)}}\\ +\overset{\text{switch}\;\text{to}\;\text{M1}\;\text{from}\;\text{M2}\;}{\overset{︷}{k_{m2m1}M2}}-\overset{\text{decay}}{\overset{︷}{\mu_{m1}M1}} \end{array}$$ (1)

$$\begin{array}{c} \frac{dM2}{dt}=\overset{\text{activation/influx}\;\text{rate}}{\overset{︷}{\frac{s_{mr}R_{M2}\left(M2\right)}{\mu_{mr}+R_{M1}\left(P,N,M1,AN\right)+R_{M2}\left(M2\right)}}}+\overset{\text{switch}\;\text{rate}\;\text{from}\;\text{M1}\;\text{per}\;\text{phagocytized}\;\text{AN}}{\overset{︷}{k_{m1m2}k_{anm1}ANf_{i}\left(M1,N\right)}}\\ -\overset{\text{switch}\;\text{from}\;\text{M2}\;\text{to}\;\text{M1}\;}{\overset{︷}{k_{m2m1}M2}}-\overset{\text{decay}}{\overset{︷}{\mu_{m2}M2}} \end{array}$$ (2)

where the activation/influx rates for M1 and M2 are given by

$$\begin{array}{c} R_{M1}=\overset{\text{activation}\;\text{by}\;P}{\overset{︷}{k_{m1p}P}}+\overset{\text{activation}\;\text{by}\;\text{byproducts}\;\text{of}\;\text{N}}{\overset{︷}{k_{m1n}N}}+\overset{\text{activation}\;\text{by}\;\text{M1s}\;\text{and}\;\text{their}\;\text{cytokines}}{\overset{︷}{k_{m1m1}M1}}\\ +\overset{\text{activation}\;\text{by}\;\text{necrotic}\;\text{AN}}{\overset{︷}{k_{m1an}\mu_{an}AN}}\\ R_{M2}=\overset{\text{activation}\;\text{by}\;\text{M2s}\;\text{and}\;\text{their}\;\text{cytokines}}{\overset{︷}{k_{m2m2}M2}}+\overset{\text{background}\;\text{anti-inflammatory}\;\text{cytokines}}{\overset{︷}{k_{c}}} \end{array}$$

Neutrophils:

$$\begin{array}{c} \frac{dN}{dt}=\overset{\text{activation}\;\text{rate}}{\overset{︷}{\frac{s_{nr}R_{N}\left(P,AN\right)}{\mu_{nr}+R_{N}\left(P,AN\right)}}}-\overset{\text{apoptosis}}{\overset{︷}{k_{an}N}} \end{array}$$ (3)

$$\begin{array}{c} \frac{dAN}{dt}=\overset{\text{apoptotis}\;\text{of}\;\text{N}}{\overset{︷}{k_{an}N}}-\overset{\text{removal}\;\text{by}\;\text{M1}}{\overset{︷}{k_{anm1}ANf_{i}\left(M1,N\right)}}-\overset{\text{removal}\;\text{by}\;\text{M2}}{\overset{︷}{k_{anm2}ANf_{i}\left(M2,N\right)}}-\overset{\text{removal}\;\text{by}\;\text{N}}{\overset{︷}{k_{ann}N}}\\ -\overset{\text{secondary}\;\text{necrosis}}{\overset{︷}{\mu_{an}AN}} \end{array}$$ (4)

where the activation rate for neutrophils is

$$\begin{array}{c} R_{N}=\overset{\text{activation}\;\text{by}\;P}{\overset{︷}{k_{np}P}}+\overset{\text{activation}\;\text{by}\;\text{necrotic}\;\text{AN}}{\overset{︷}{k_{nan}\mu_{an}AN}} \end{array}$$

Inflammatory Stimulus:

$$\begin{array}{c} \frac{dP}{dt}=\overset{\text{logistic}\;\text{broth-dependent}\;\text{growth}}{\overset{︷}{k_{pg}P(1-\frac{P}{P_{\infty }+B})}}-\overset{\text{removal}\;\text{by}\;\text{N}}{\overset{︷}{k_{pn}PN}}-\overset{\text{removal}\;\text{by}\;\text{M1}}{\overset{︷}{k_{pm}Pf_{i}\left(M1,N\right)}}\\ -\overset{\text{removal}\;\text{by}\;\text{M2}}{\overset{︷}{k_{pm}Pf_{i}\left(M2,N\right)}} \end{array}$$ (5)

$$\begin{array}{c} \frac{dB}{dt}=\overset{\text{consumption}\;\text{by}\;\text{P}}{\overset{︷}{-k_{b}BP}} \end{array}$$ (6)

Inhibition function:

$$\begin{array}{c} f_{i}\left(x,N\right)=\frac{x}{1+{\left(\frac{N}{n_{\infty }}\right)}^{2}} \end{array}$$

The healthy peritoneal cavity is impermeable and is assumed to be nearly sterile prior to inflammatory stimulus, with very low levels of pathogen, and so has no immune cell influx. Therefore, all of our immune cell variables have an initial condition of zero. The injection of nutrient broth is assumed to stimulate a very rapid increase in pathogen growth that quickly subsides as broth is consumed and pathogen is removed by macrophages and neutrophils. This brief spike in pathogen modeled by Eqs 5 and 6 initiates the subsequent immune cell response.

As in Cooper et al. [23], immune cells are assumed to activate and influx into the local environment rapidly compared to other dynamics, so the quasi-steady state assumption is used. This gives rise to Michaelis-Menten type activation and influx terms in Eqs 1–3. In addition, we do not explicitly model cytokines but instead allow the production of immune cells to act as an indicator of associated cytokine level.

Resting neutrophils are the first immune cells to arrive at the site of infection, rapidly becoming activated by pathogen and the debris formed by apoptotic neutrophils at the rate *R*_*N*_(*P*, *AN*). As neutrophils become laden with bacteria, they undergo apoptosis at rate *k*_*an*_. Apoptotic neutrophils are then removed by M1s at rate *k*_*anm*1_, M2s at rate *k*_*anm*2_, and by active neutrophils at rate *k*_*ann*_. We have chosen *k*_*ann*_ to be much smaller than both *k*_*anm*1_ and *k*_*anm*2_ as appropriate for the case when both macrophages and neutrophils are present, but in the absence of macrophages, the clearance of apoptotic cells by neutrophils may take on greater importance [44, 45]. Apoptotic neutrophils that are not cleared undergo secondary necrosis at rate *μ*_*an*_, contributing to the positive feedback described in the neutrophil activation term *R*_*N*_.

Resting monocytes (*M*_*R*_) are next to arrive. The majority of these first monocytes differentiate to an inflammatory M1 phenotype in response to pathogen, byproducts of neutrophils, M1s and their cytokines, and cytokines spilled by necrotic apoptotic neutrophils at rate *R*_*M*1_(*P*, *N*, *M*1, *AN*). Background levels of anti-inflammatory cytokines, *k*_*c*_ (related to the anti-inflammatory source term in Reynolds et al. [14]), cause a small portion of monocytes to differentiate to an M2 phenotype. Intrinsic decay is assumed to occur at rate *μ*_*m*1_ in M1s and at rate *μ*_*m*2_ in M2s. M1s are assumed to be able to switch to M2s at rate *k*_*m*1*m*2_, and this switch is assumed to be promoted by the phagocytosis of apoptotic cells [1, 3, 42, 46]. Plasticity of macrophage phenotype is not fully understood, therefore, we allow for the possibility of a transition from M2 to M1 in Eq 1 at rate *k*_*m*2*m*1_ as well. Late arriving monocytes are assumed to be able to activate to the M2 phenotype in response to anti-inflammatory cytokines produced by M2s at rate *R*_*M*2_(*M*2).

The inhibition term *f*_*i*_(*x*, *N*) models the inhibition of macrophage activity by neutrophils due to oxidation of the environment. The same parameter, *n*_∞_, is used to determine the level at which the presence of neutrophils inhibit the macrophages regardless of phenotype (M1 or M2) and what they are phagocytosing (pathogen or apoptotic neutrophils). This is due to the simplifying assumption that all macrophages are inhibited the same by the oxidative stress in the local environment.

### Parameter estimation

Cell count data is given in units of 10^7^ cells. The model given by Eqs 1–6 was fit to experimental data using the trust region method within PottersWheel, a MATLAB toolbox for parameter estimation [47]. The trust region approach uses the *lsqnonlin* algorithm of MATLAB’s optimization toolbox, which allows for the specification of bounds on the parameter space to be searched. Bounds for each parameter are given in Table 1.

The fitting procedure was then performed iteratively via weighted least squares with merit function

$$\begin{array}{c} \chi^{2}\left(\vec{p}\right)=\sum_{i=1}^{n}(\frac{y_{i}-y\left(t_{i};\vec{p}\right)}{\sigma_{\bar{i}}}{)}^{2} \end{array}$$ (7)

with $\vec{p}$ the vector of estimated parameters, *y*_*i*_ the observations, $y(t_{i};\vec{p})$ the model predictions given the parameter estimates, $\sigma_{\bar{i}}$ the standard errors, and *n* equal to the total number of observations over all response variables. Minimizing $\chi^{2}\left(\vec{p}\right)/2$ is equivalent to maximizing the log-likelihood

$$\begin{array}{c} \log L\left(\vec{p}|y_{data}\right)=-\sum_{i}\frac{{\left(y_{i}^{data}-y_{i}^{model}\right)}^{2}}{2\sigma_{i}^{2}}-N\log\sqrt[{}]{2\pi }-\sum_{i}\log\sigma_{i} \end{array}$$

since only the first term is parameter-dependant [47].

Fitting was performed in logarithmic parameter space since some parameter bounds span several orders of magnitude. This local optimization routine seeks parameters that minimize the sum of squared errors between the data and model predictions while accounting for variance. Since each observable *N*, *M*1, and *M*2 has high standard deviations for measurements taken at time points near the maximum, weighting by these standard deviations would result in compliance with many models. We chose instead to use error model *σ*_*i*_ = 0.05*y*_*i*_ + 0.1max(*y*), assuming 5% uncertainty at each time point and 10% overall uncertainty relative to the maximum of each observable.

At each step of the fitting process, parameter estimations were performed iteratively to ensure minimization of the merit function. Results at each step were analyzed to determine free and fixed parameters and to narrow the search for an identifiable subset of parameters as described in the Results section.

### Goodness-of-fit measures

Under the assumption that residuals between the data and model predictions are Gaussian distributed, the log-likelihood is distributed like a *χ*^2^ distribution with *N* − *M* degrees of freedom, with *N* data points and *M* parameters being estimated [47]. PottersWheel calculates a *χ*^2^ p-value after each fit for the null hypothesis that (1) the model sufficiently explains the data, (2) true standard deviations do not exceed standard deviation estimates, and (3) the residuals are normally distributed [47].

PottersWheel also calculates the Akaike Information Criterion AIC=−2logL+2p for a model with *p* parameters [48]. Given two models under consideration, the one with the lowest *AIC* value is preferred.

## Results

With the general model developed, we next estimate model parameters, analyze sensitivity of model characteristics to perturbations in the parameters and, finally, predict changes in neutrophil and macrophage behavior in response to parameter variations.

### Determination of an identifiable subset of model parameters for estimation

**Fig 3:**
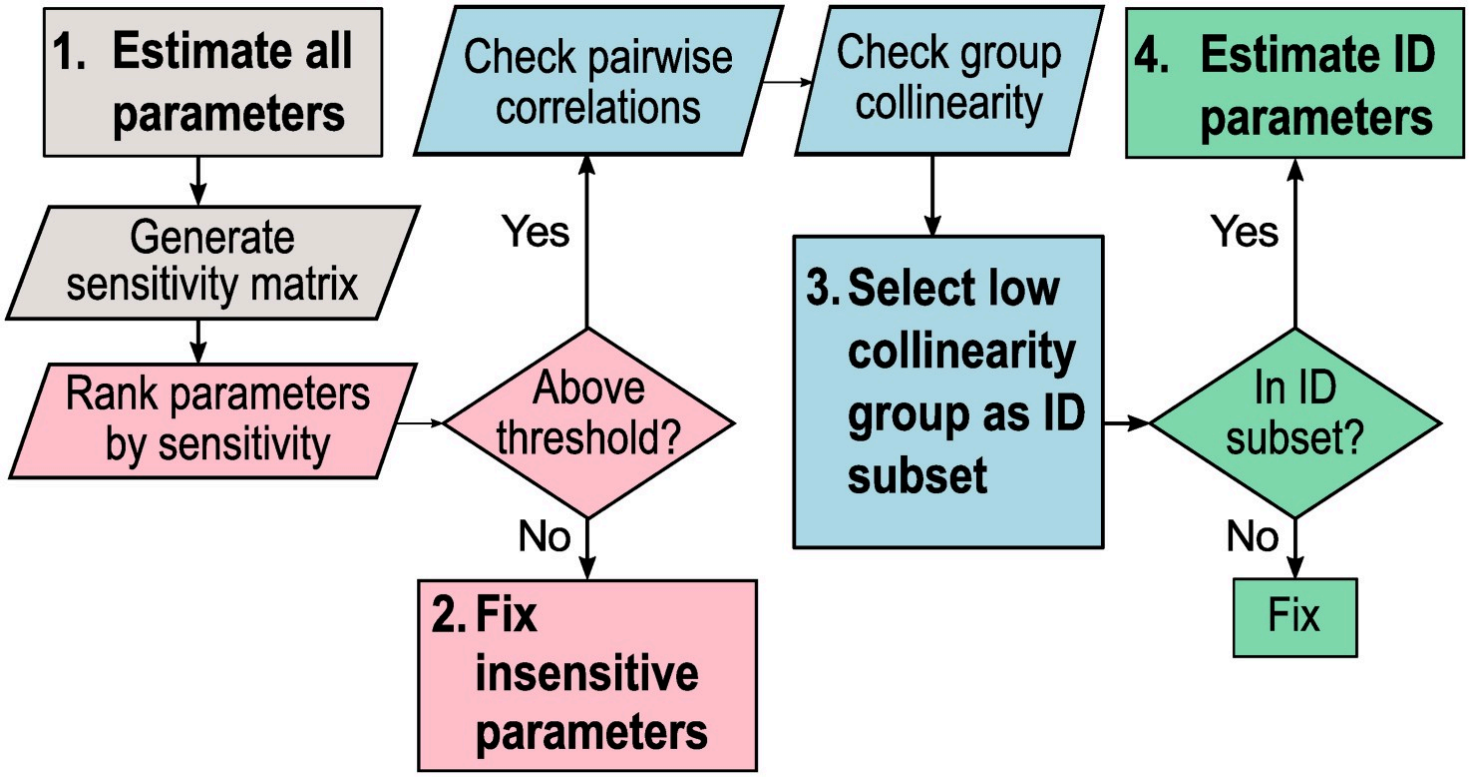
Steps to estimate an identifiable subset of parameters. Step 1 (gray): estimate all parameters and generate a discretized sensitivity matrix from the fitted model. Step 2 (pink): Fix parameters that fall below a determined sensitivity threshold. Step 3 (blue): Select one group of low collinearity (identifiable) parameters. Step 4 (green): Estimate the chosen identifiable subset and fix all other parameters.

Structural identifiability (SI) is a prerequisite for model prediction [49], while numerical or practical identifiability is required to determine confidence intervals around parameter estimates and ensure that the connection between the dynamic model and the data model is sufficiently strong for prediction. Determining which parameters can be uniquely determined, or at least limited to a finite range of possible values, is also a critical step in informing further experimentation. This process includes selecting parameters that significantly impact model outputs as well as defining interactions between parameters that can influence parameter estimates obtained during fitting. In this section, we analyze local parameter identifiability as outlined in the steps below and in Fig 3:

1. Estimate all parameters.
  - Use the fitted model to generate the discretized sensitivity matrix *S*.
2. Fix insensitive parameters.
  - Use *S* to rank parameters by sensitivity.
  - Set a threshold such that parameters with sensitivity below the threshold (insensitive) are fixed and parameters with sensitivity above the threshold (sensitive) are analyzed in Step 3.
3. Select low collinearity group of parameters as identifiable (ID) subset.
  - Check for pairwise correlations between parameters by deriving an approximate correlation matrix from *S*.
  - Check for collinearity between groups of parameters with a collinearity index (*CI*) measure. Set a threshold such that groups of parameters with *CI* above the threshold are considered collinear. Groups of parameters with *CI* below the threshold are considered identifiable subsets.
4. Estimate identifiable (ID) subset of parameters.
  - One identifiable subset of parameters is selected to be estimated.
  - The remaining parameters are fixed.

Model parameters were estimated using a maximum likelihood equivalent criterion and trust region search algorithm as described (see Materials and methods). Since reducing parameters to be estimated can be considered a form of model reduction [50], we refer to our final model with 6 estimated parameters as the “identifiable” model versus the “full” model with all 24 parameters estimated in the comparisons below.

First, we performed parameter estimation on the full model. For all three observable model outputs (*N*, *M*1, and *M*2) sampled at 10 time points with 24 model parameters, a 30 × 24 discretized sensitivity matrix *S* is produced. To test structural identifiability of the model *a posteriori*, we generated these matrices at a variety of locations in parameter space within the bounds given in Table 1 and found the rank and the singular values for each. Since each of these matrices was determined to have full column rank and no zero singular values, we concluded that the model is locally SI [51] within the bounds we had set for parameter estimation.

Next, we ranked the impact of each parameter on all three observable model outputs (*N*, *M*1, and *M*2) by calculating a root mean square sensitivity measure, as defined in Brun et al. [34], for each column *j* of the normalized sensitivity matrix as

$$\begin{array}{c} RMS_{j}=\sqrt[{}]{\frac{1}{n}\sum_{i=1}^{n}(\frac{p_{j}}{y_{i}}\frac{\partial y_{i}}{\partial p_{j}}{)}^{2}}. \end{array}$$

**Fig 4:**
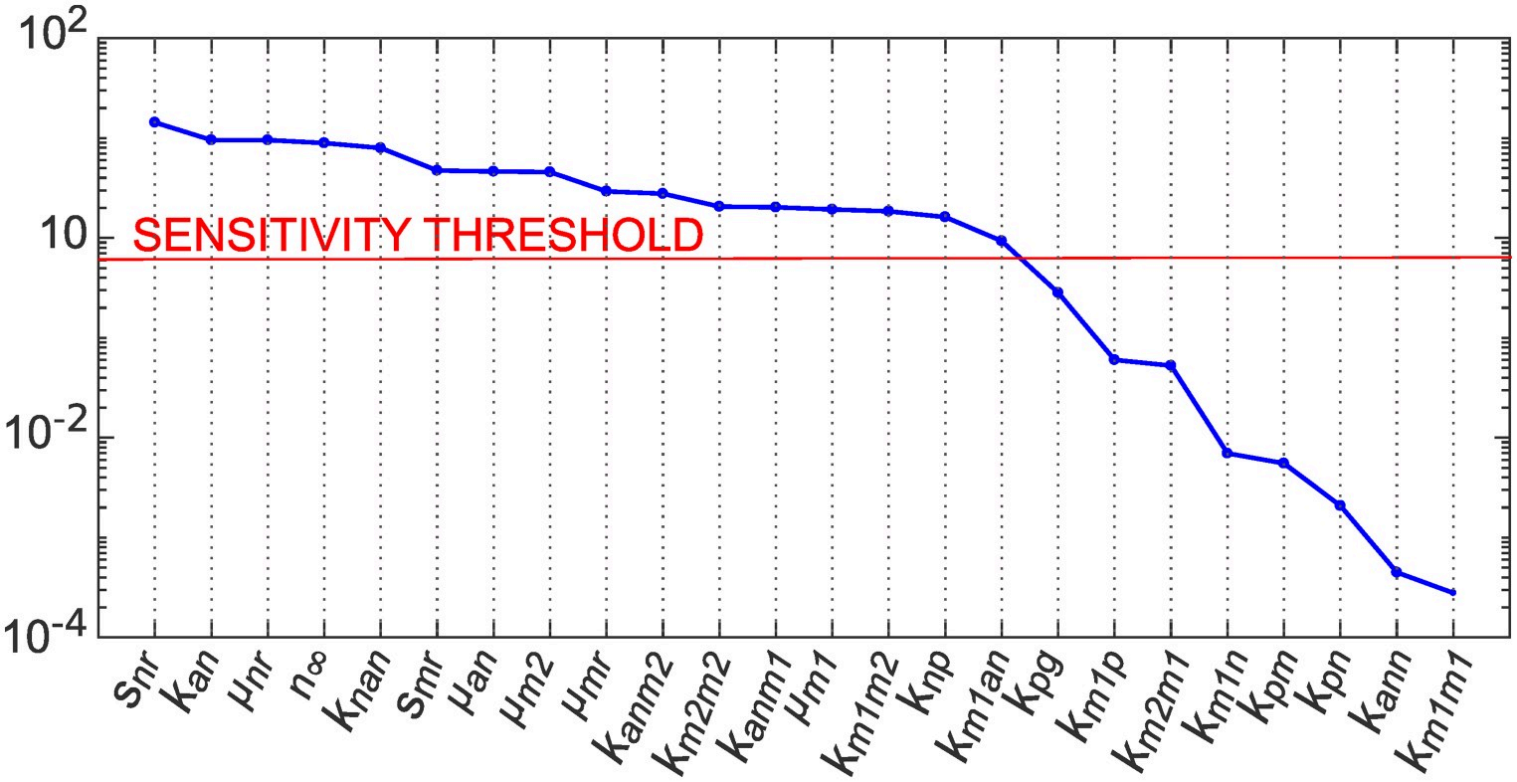
Parameter importance ranking (*RMS*) for full and identifiable model. We ranked the impact of each parameter on all three observable model outputs (*N*, *M*1, and *M*2) by calculating a root mean square sensitivity measure, as defined in Brun et al. [34]. The sensitivity threshold was set at 5% of the maximum *RMS* value calculated over all parameters. Eight parameters in the full model were thus deemed insensitive and fixed in step 2 of our identifiability analysis. The inset plot shows *RMS* values for the identifiable model.

Parameter *j* is deemed insensitive if *RMS*_*j*_ is less than 5% of the value of the maximum *RMS* value calculated over all parameters. By this measure, 8 parameters were deemed insensitive, as shown in Fig 4, and fixed at their nominal values.

We had determined that all singular values were greater than zero, indicating SI, but only 6 of the 24 singular values obtained had values with order of magnitude greater than zero. If we consider the very small singular values essentially zero for the purpose of rank calculation (in order to reduce problems with numerical identifiability) this gives rank(*S*) = 6, and since rank(*S*) can be used to identify the number of parameters that can be included in an identifiable subset [36, 50], a subset of size 6 is suggested. The parameter estimation problem was therefore reduced to finding identifiable subsets of size 6 out of the 16 sensitive parameters.

**Fig 5:**
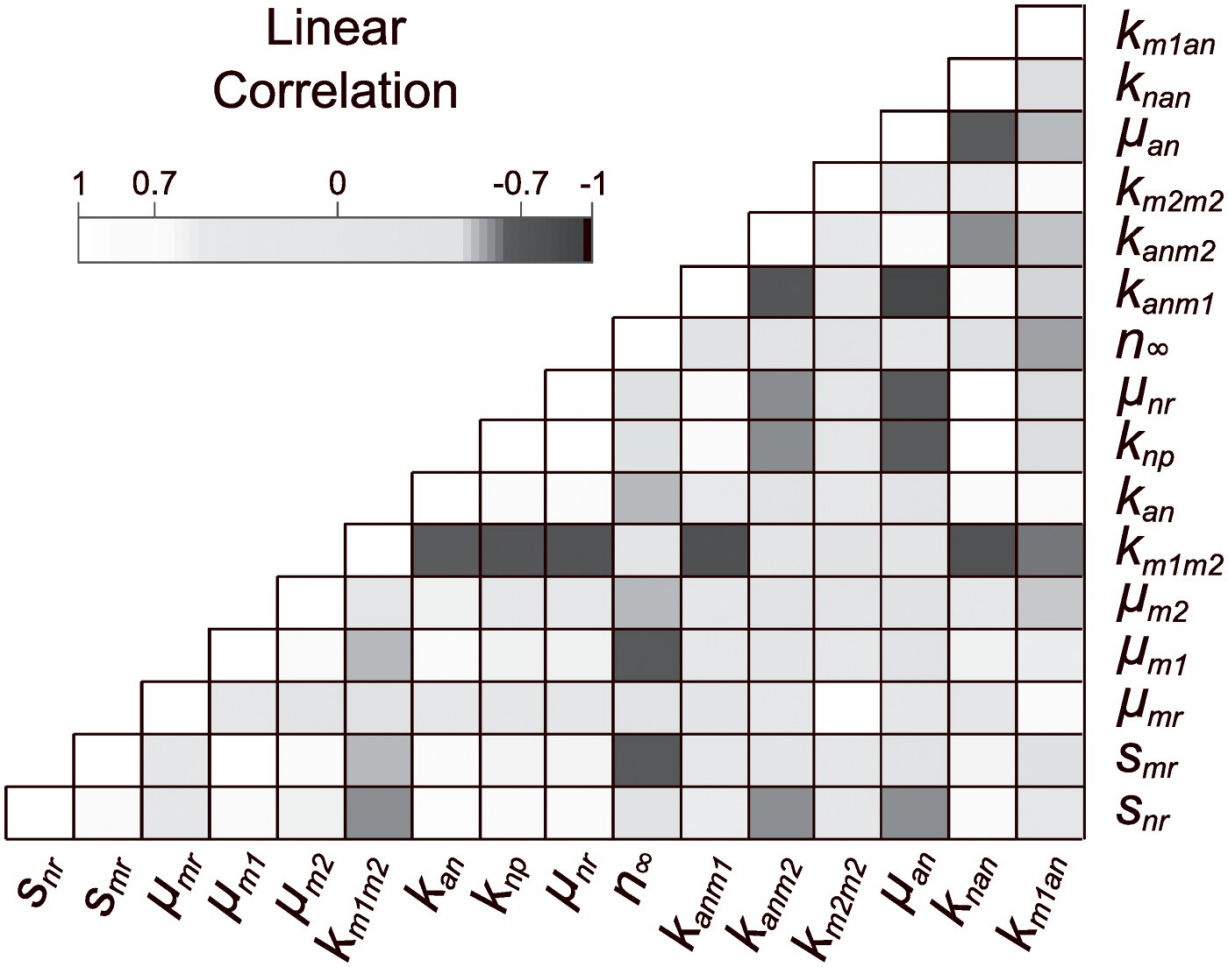
Correlation matrix plot for the full model. An approximate correlation matrix was obtained from the Fisher Information Matrix for the sensitive subset of parameters and used to visualize correlations. There are many significant linear correlations (greater than 0.7) between sensitive parameters that appear as black or white squares on the off diagonal.

The estimated correlation matrix for the sensitive subset of parameters, shown in Fig 5, shows a large number of dependencies between pairs of parameters. Effects of nearly linearly dependent parameters on output are pairwise indistinguishable and cannot be reliably estimated, due to compensating effects by changes in other parameters in the group. In addition to discovering pairwise parameter relationships, we sought a minimally correlated group of 6 parameters. A measure that applies to parameter subsets of any size is the collinearity index defined by Brun et al. [34] as

$$\begin{array}{c} CI=\frac{1}{{\sqrt{\lambda }}_{k}} \end{array}$$

where λ_*k*_ is the smallest eigenvalue of ${\bar{S}}_{k}^{T}{\bar{S}}_{k}$ and $\bar{S}$ is a submatrix of *S* containing the sensitivity vectors for parameters in subset *K*. In practical terms, changes in model output caused by a change in parameter *p*_*j*_ can be compensated for by other parameters by up to $\frac{1}{CI}$ (e.g., for *CI* = 20 a change in output caused by a change in *p*_*j*_ can be compensated for up to 5% by other parameters in subset *K*) [34]. A cutoff of *CI* = 20 was used to select subsets of parameters with low collinearity.

Collinearity indices were calculated for parameter subsets of increasing size as described in Brun et al. [34], using code in the VisId MATLAB toolbox [35]. Thirteen parameter pairs that were found highly correlated by this measure are shown in Table 2; others are not shown due to the large number of collinear groups (for example, there were 68 highly collinear parameter subsets of size 3). No subsets of size greater than 6 met our criteria for low collinearity between parameters. In all, 25 parameter subsets of size 6 met our criteria, involving 10 different parameters (shown in Table 3).

**Table 2:** Pairwise collinearity indices.

Parameter Pair	*CI*
*k*_*m*1*m*2_, *k*_*anm*1_	67.98
*s*_*nr*_, *u*_*nr*_	47.04
*s*_*nr*_, *k*_*nan*_	46.16
*u*_*nr*_, *k*_*nan*_	43.50
*k*_*an*_, *k*_*nan*_	28.28
*s*_*nr*_, *k*_*an*_	26.79
*k*_*an*_, *u*_*nr*_	25.79
*u*_*nr*_, *u*_*an*_	25.13
*n*_∞_, *k*_*nan*_	24.84
*k*_*an*_, *n*_∞_	24.20
*s*_*nr*_, *u*_*an*_	24.14
*u*_*an*_, *k*_*nan*_	20.77
*s*_*nr*_, *n*_∞_	20.16

Pairs of parameters were considered collinear (highly correlated) if *CI* > 20.

**Table 3:** All identifiable parameter subsets of size 6.

Parameter group	Collinearity Index
*s*_*nr*_, *s*_*mr*_, *μ*_*m*1_, *k*_*m*1*m*2_, *k*_*np*_, *k*_*anm*2_	18.492
*s*_*nr*_, *s*_*mr*_, *μ*_*m*1_, *k*_*np*_, *k*_*anm*1_, *k*_*anm*2_	18.915
*s*_*nr*_, *s*_*mr*_, *μ*_*m*2_, *k*_*m*1*m*2_, *k*_*np*_, *k*_*anm*2_	18.726
*s*_*nr*_, *s*_*mr*_, *μ*_*m*2_, *k*_*np*_, *k*_*anm*1_, *k*_*anm*2_	19.281
*s*_*nr*_, *s*_*mr*_, *k*_*m*1*m*2_, *k*_*np*_, *k*_*anm*2_, *k*_*m*2*m*2_	18.197
*s*_*nr*_, *s*_*mr*_, *k*_*m*1*m*2_, *k*_*np*_, *k*_*anm*2_, *k*_*m*1*an*_	19.170
*s*_*nr*_, *s*_*mr*_, *k*_*np*_, *k*_*anm*1_, *k*_*anm*2_, *k*_*m*2*m*2_	18.562
*s*_*nr*_, *s*_*mr*_, *k*_*np*_, *k*_*anm*1_, *k*_*anm*2_, *k*_*m*1*an*_	19.815
*s*_*nr*_, *μ*_*mr*_, *μ*_*m*1_, *k*_*m*1*m*2_, *k*_*np*_, *k*_*anm*2_	18.009
*s*_*nr*_, *μ*_*mr*_, *μ*_*m*1_, *k*_*np*_, *k*_*anm*1_, *k*_*anm*2_	18.311
*s*_*nr*_, *μ*_*mr*_, *μ*_*m*1_, *k*_*np*_, *k*_*anm*2_, *k*_*m*1*an*_	19.060
*s*_*nr*_, *μ*_*mr*_, *k*_*m*1*m*2_, *k*_*np*_, *k*_*anm*2_, *k*_*m*2*m*2_	18.606
*s*_*nr*_, *μ*_*mr*_, *k*_*m*1*m*2_, *k*_*np*_, *k*_*anm*2_, *k*_*m*1*an*_	18.323
*s*_*nr*_, *μ*_*mr*_, *k*_*np*_, *k*_*anm*1_, *k*_*anm*2_, *k*_*m*2*m*2_	19.032
*s*_*nr*_, *μ*_*mr*_, *k*_*np*_, *k*_*anm*1_, *k*_*anm*2_, *k*_*m*1*an*_	18.741
*s*_*nr*_, *μ*_*m*1_, *μ*_*m*2_, *k*_*m*1*m*2_, *k*_*np*_, *k*_*anm*2_	18.370
*s*_*nr*_, *μ*_*m*1_, *μ*_*m*2_, *k*_*np*_, *k*_*anm*1_, *k*_*anm*2_	18.800
*s*_*nr*_, *μ*_*m*1_, *μ*_*m*2_, *k*_*np*_, *k*_*anm*2_, *k*_*m*1*an*_	19.984
*s*_*nr*_, *μ*_*m*1_, *k*_*m*1*m*2_, *k*_*np*_, *k*_*anm*2_, *k*_*m*2*m*2_	18.207
*s*_*nr*_, *μ*_*m*1_, *k*_*np*_, *k*_*anm*1_, *k*_*anm*2_, *k*_*m*2*m*2_	18.567
*s*_*nr*_, *μ*_*m*1_, *k*_*np*_, *k*_*anm*2_, *k*_*m*2*m*2_, *k*_*m*1*an*_	19.251
*s*_*nr*_, *μ*_*m*2_, *k*_*m*1*m*2_, *k*_*np*_, *k*_*anm*2_, *k*_*m*1*an*_	18.610
*s*_*nr*_, *μ*_*m*2_, *k*_*np*_, *k*_*anm*1_, *k*_*anm*2_, *k*_*m*1*an*_	19.121
*s*_*nr*_, *k*_*m*1*m*2_, *k*_*np*_, *k*_*anm*2_, *k*_*m*2*m*2_, *k*_*m*1*an*_	18.364
*s*_*nr*_, *k*_*np*_, *k*_*anm*1_, *k*_*anm*2_, *k*_*m*2*m*2_, *k*_*m*1*an*_	18.771

A subset of sensitive parameters was considered identifiable if its collinearity index was below 20. Of these twenty-five identifiable subsets of size 6 (generated from 10 parameters), we chose one subset to estimate given in Table 4. With our choice, we sought to both minimize the *CI* and maximize the sum of the *RMS* sensitivity measures over all of the parameters in a subset containing parameters that may be reasonably estimated from currently available data and that we hope to vary in future simulated experiments.

In selecting one of these parameter subsets to be estimated in an identifiable model, we considered several factors. First, from a practical standpoint, it was desirable to choose parameters that may be reasonably estimated from currently available data and also that we hope to vary in future simulated experiments. Next, we sought to both minimize the *CI* and maximize the sum of the *RMS* sensitivity measures over all of the parameters in the subset. Minimizing the *CI* reduces the likelihood of parameter dependencies interfering with optimization, while choosing the subset with the most sensitive parameters should require the smallest adjustment to their values [50].

**Fig 6:**
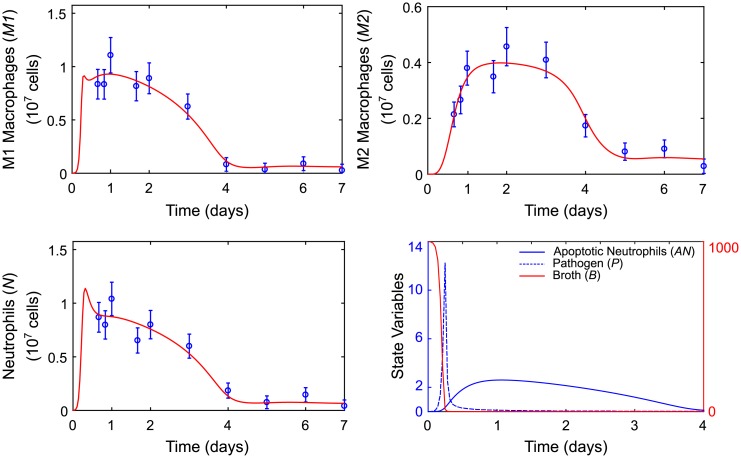
Model predictions for the identifiable model. Model response variable predictions for M1 macrophage (*M*1), M2 macrophage (*M*2), and neutrophil (*N*) counts are plotted versus mean observed values and standard errors. Model state variable predictions for levels of pathogen (*P*) and nutrient (*B*) and apoptotic neutrophil (*AN*) counts are plotted on the same axis. The blue axis applies to pathogen and apoptotic neutrophils. The red axis applies to nutrient broth.

The chosen identifiable subset of 6 parameters is shown in Table 4 along with pointwise 95% confidence intervals calculated based on the approximate Hessian matrix of the objective function given in Eq 7, as described in Maiwald et al. [47]. The fit of the identifiable model to M1, M2, and neutrophil data along with state variable predictions for pathogen, nutrient broth, and apoptotic neutrophils are shown in Fig 6. A plot of the differences between model predictions and observations is available in the Supporting Information.

**Table 4:** Parameter values and 95% pointwise confidence intervals for identifiable model.

Parameter	Estimate	95% CI
*μ*_*m*1_	6.83	(5.45, 8.54)
*k*_*m*1*m*2_	8.62	(5.56, 13.4)
*k*_*m*2*m*2_	1.59	(0.86, 1.96)
*s*_*nr*_	16.4	(16.0, 16.8)
*k*_*np*_	3.10	(1.68, 5.65)
*k*_*anm*2_	91.0	(66.4, 125)

Remaining parameters were fixed at values given in Table 1.

### Goodness-of-fit

The full model, with 24 parameters estimated, and the identifiable model, with 6 parameters estimated, are compared with respect to goodness-of-fit using the Akaike information criterion (AIC) and *χ*^2^ test (see Methods) in Table 5. By these measures, the data is best explained by the identifiable model even though the difference in *χ*^2^ metric value between models is small. There is close agreement between model predictions and observations achieved with our obtained parameters, however, we remark that there is some dependency between fixed and estimated parameters and that there are inherent limitations in estimating parameters with limited experimental data. Therefore, these estimates should be taken as conditional, and we can determine which fixed parameters they may be conditioned on by viewing the profile likelihood [37, 52, 53].

**Table 5:** Goodness-of-fit statistics.

	*n*_*p*_	*χ*^2^	p-value	AIC
Full model	24	19.325	0.003	122.462
Identifiable model	6	15.473	0.906	82.61

In the full model, 24 parameters were estimated. After identifiability analysis, estimated parameters were reduced to 6 and the remaining parameters were fixed prior to fitting. The reduction in estimated parameters improved the weighted least squares merit function value (*χ*^2^), increased p-value on a *χ*^2^ test indicating that the identifiable model sufficiently explains the data, and lowered the estimated amount of information lost between the model and the data by the Aikake Information Criterion (AIC) measure.

The profile likelihood approach for analyzing identifiability fixes a parameter *p*_*i*_ at values over a specified range, re-estimating all other parameters at each point [52]:

$$\begin{array}{c} \chi_{PL}^{2}\left(p_{i}\right)=\min_{p_{j}\neq i}[\chi^{2}\left(p\right)]. \end{array}$$

Using the profile likelihood, it is possible to trace out the functional form of identifiable combinations of parameters, and this information can be used in re-parametrization [36, 37]. However, this requires reducing extra degrees of freedom in the estimated parameters in order to avoid compensation effects. [37]. Even with collinearities present, it is possible to get an idea of compensation effects between parameters during fitting by observing how estimated parameters change over the profiled parameter. This can be important in determining whether estimated parameters are conditional on parameters that were fixed prior to fitting [34]. We have plotted the profile likelihoods of parameters in the identifiable subset versus other parameters that change significantly over the profile likelihood (see Supporting Information).

### Sensitivity analysis

**Fig 7:**
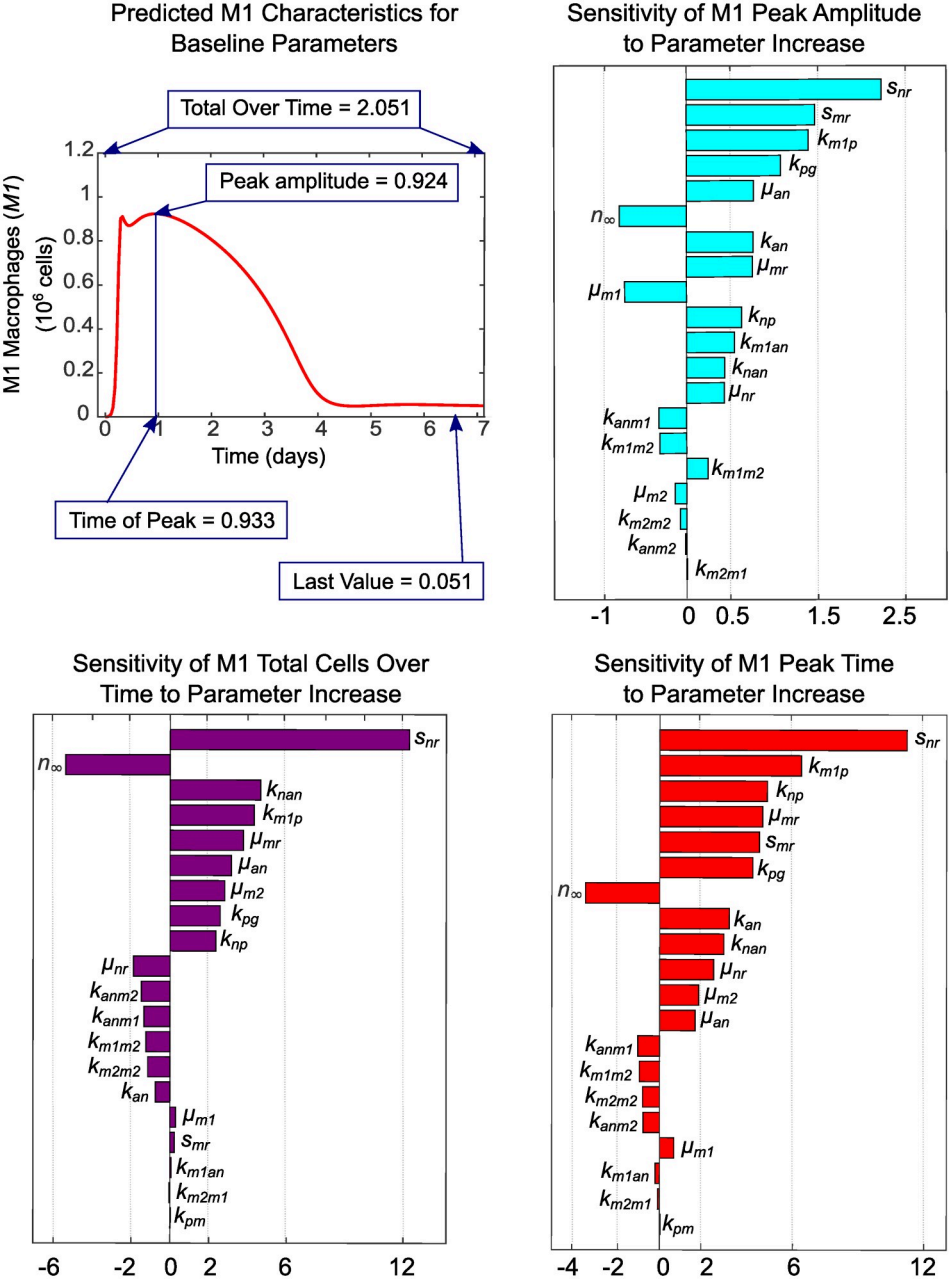
Baseline characteristics for M1 and sensitivity of characteristics to parameter variations. The M1 transient curve and its characteristics are plotted for the baseline parameter values given in Tables 1 and 4. Parameter sensitivity plots show the effects on M1 characteristics of varying model parameters one-at-a-time by a factor of 1.001 of its baseline value while holding all other parameters at their baseline values. Insensitive parameters, which have zero sensitivity for all characteristics, are not shown.

**Fig 8:**
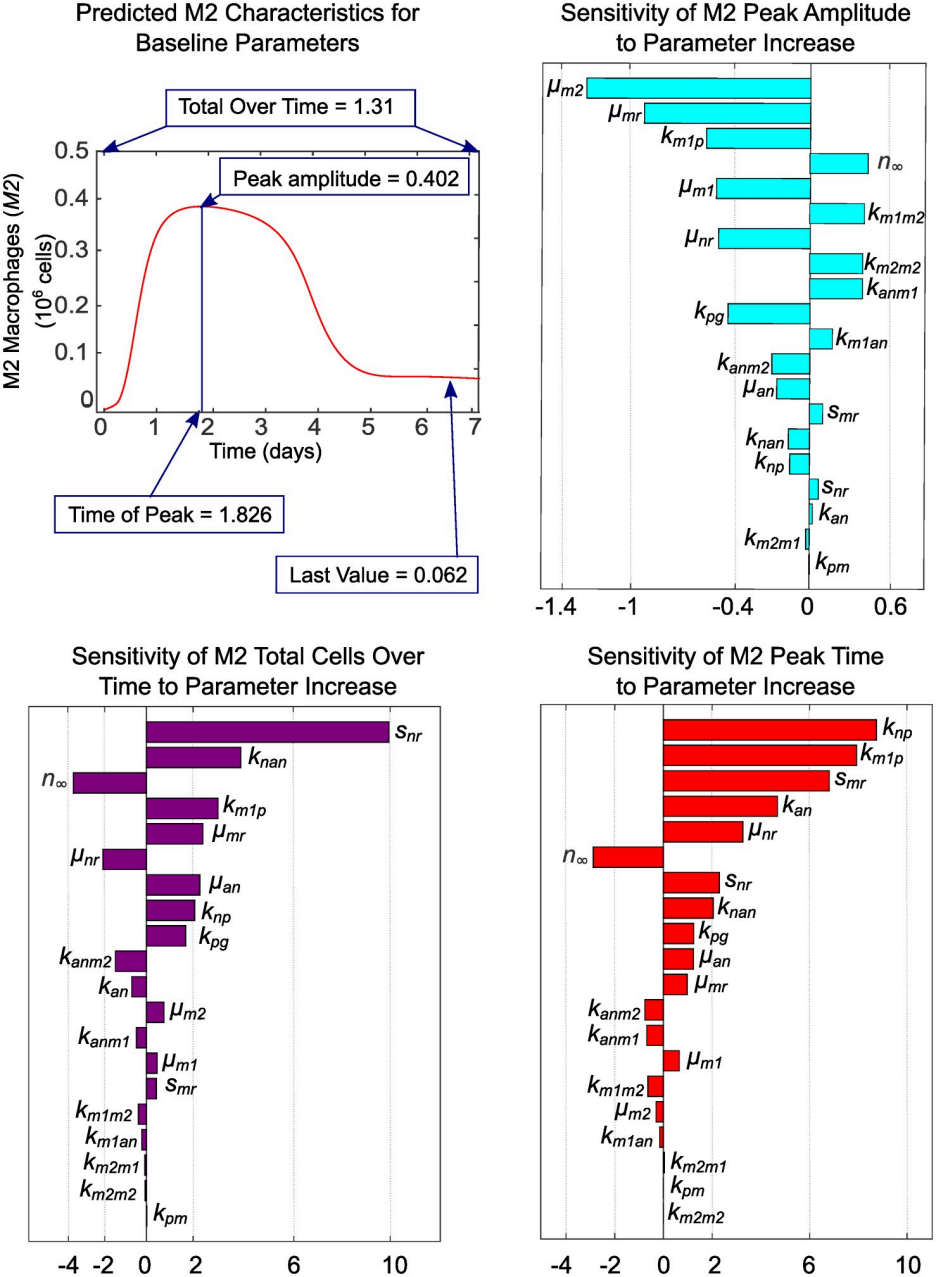
Baseline characteristics for M2 and sensitivity of characteristics to parameter variations. The M2 transient curve and its characteristics are plotted for baseline parameter values given in Tables 1 and 4. Parameter sensitivity plots show the effects on M2 characteristics of varying model parameters one-at-a-time by a factor of 1.001 of its baseline value while holding all other parameters at their baseline values. Insensitive parameters, which have zero sensitivity for all characteristics, are not shown.

The impact that both fixed and estimated parameters have on predictions for *M*1 and *M*2 macrophages was analyzed with one-at-a-time sensitivity analysis. We focused our analysis on these two observable outputs since our goal is to identify drivers of population level phenotype switch in macrophages. In applying this method, we increased each parameter by a factor of 1.001 of its baseline value while holding all other parameters at their baseline values to determine the effects on the M1 and M2 characteristics shown in Figs 7 and 8. The sensitivity of characteristic *f* with respect to parameter *p* is then estimated as *s* = (*f*(1.001 * *p*) − *f*(*p*))/(1.001 * *p* − *p*) * *p*/*f* using the PottersWheel MATLAB toolbox [47]. The parameter is then reset to its baseline value and the process is repeated for the next parameter, until sensitivity of all parameters is analyzed. Baseline values for parameters that were fixed during fitting are given in Table 1 and baseline estimated parameter values are given in Table 4. Baseline characteristics of each cell type are shown in Figs 7 and 8, along with sensitivities of each characteristic to variations in each parameter. Since parameters are varied individually, this analysis does not take into account interactions between variables that may influence model results in unexpected ways if more than one parameter is varied simultaneously. Taken with the above caution, however, we can gain some insight into which factors may drive macrophage phenotype balance.

The most influential parameters on M1 behavior are *s*_*nr*_ and *s*_*mr*_ (availability of resting neutrophils and monocytes), *k*_*pg*_ (behavior of inflammatory stimulus), *k*_*m*1*p*_ and *k*_*np*_ (response of M1s and neutrophils to inflammatory stimulus), and *u*_*an*_ (rate of secondary necrosis of neutrophils). In the present context, M1s are primarily activated by initial inflammatory stimulus and necrosis of apoptotic neutrophils that have not been phagocytosed. This supports the hypothesis that effective clearance of apoptotic cells is important in the resolution of inflammation [1, 40, 46, 54–59]. If our parameter estimates had been obtained by fitting to data from chronic inflammation, feedback from existing M1s and the pro-inflammatory byproducts of existing neutrophils would likely be greater contributors to M1 response. Negatively related to magnitude of M1 response are parameters *μ*_*m*1_ (decay or efflux rate of M1s) and *n*_∞_ (the level of neutrophils required to inhibit macrophage activity by 50%). As the threshold for inhibition of M1s increases, the magnitude of the M1 population decreases because less M1s are required to mount an adequate response.

The importance of neutrophils and neutrophil apoptosis in mounting a timely and sufficient M2 response is evidenced by the high sensitivity of M2 peak timing and amplitude to neutrophil-associated parameters *s*_*nr*_, *u*_*an*_, *k*_*an*_, *k*_*np*_, *u*_*nr*_, *n*_∞_, *k*_*anm*1_, *k*_*anm*2_, and *k*_*nan*_. The magnitude of the M2 population peak is also strongly positively associated with *k*_*m*1*m*2_ (switch rate from M1s) and *k*_*m*2*m*2_ (feedback from existing M2s). Increasing rates of decay or efflux for resting monocytes (*μ*_*mr*_) and resting neutrophils (*u*_*nr*_) diminishes M2 population magnitude, as does reduced M1 activation by pathogen (*k*_*m*1*p*_), indicating M2 dependence on the population size of other immune cells.

### Simulations

Our objective in this work is to identify key drivers of macrophage phenotype balance during the inflammatory response, in order to identify potential clinical targets. Therefore we now perturb parameters from fitted values in order to view effects on model behavior and simulate therapeutic targeting of macrophages for intervention in the early inflammatory process critical to disease progression, as has been proposed [60–62].

One proposed strategy to dampen inflammation is to directly polarize M1 macrophages to an M2 phenotype [60]. To evaluate the effects of varying the transition rate of M1 to M2, we varied parameter *k*_*m*1*m*2_ over 10 linearly spaced values within a factor of 1 ±.3 of its baseline value. The model predicts that increasing *k*_*m*1*m*2_ has a small effect on M1 magnitude of response while increasing the magnitude of M2 response, which is expected. However, the time course of both macrophage populations is predicted to be shortened due to a higher transition rate; whether this results in faster resolution of inflammation or an insufficient M2 population for a subsequent proliferation or repair phase may depend on the nature and magnitude of the inflammatory stimulus.

**Fig 9:**
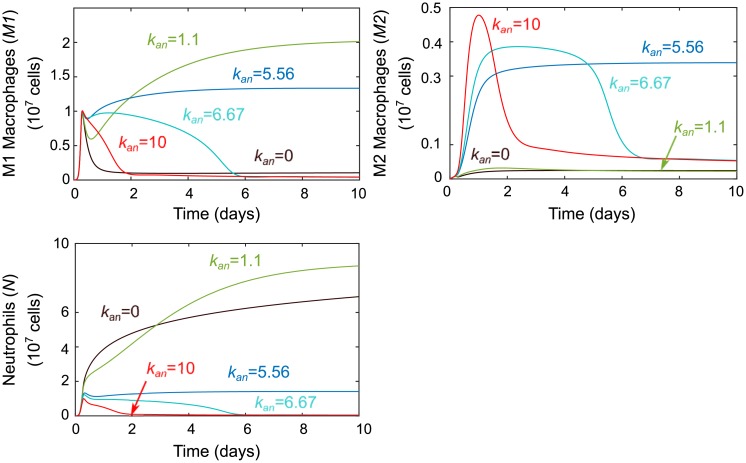
Results of perturbations in parameter *k*_*an*_. Parameter *k*_*an*_, which models the rate of neutrophil apoptosis, was varied around its baseline value of *k*_*an*_ = 7.108. The effects of variations on M1, M2, and neutrophils are shown. Values lower than baseline lead to a sustained inflammatory response from all immune cells while higher values shorten the time course of each.

Next, we simulated a change in the apoptosis rate of neutrophils, *k*_*an*_, based on our hypothesis that efferocytosis (phagocytic removal of apoptotic and necrotic cells) is a key driver of macrophage phenotype change and that this requires a sufficiently sized population of apoptotic cells [1, 3, 40–42]. Dysregulation of neutrophil population level and turnover is known to be a direct contributor to human inflammatory and autoimmune diseases such as coronary artery disease, rheumatoid arthritis, acute arterial occlusions, gout, asthma, and many others [63, 64]. Macrophages themselves are known to modulate neutrophil lifespan by releasing cytokines that can delay apoptosis [65] and some microbial pathogens delay or accelerate neutrophil apoptosis to promote their own growth [63]. From the results in Fig 9, we note that modulating the size *k*_*an*_ has some interesting effects. In the biologically unlikely case where *k*_*an*_ = 0 and there is no population of apoptotic neutrophils available for efferocytosis, neutrophils remain the dominant immune cell. For low values of *k*_*an*_, sustained inflammation appears to be the result of too many inflammatory neutrophil byproducts and the low M2 population levels. Midrange *k*_*an*_ values were determined during fitting to produce a normal response, while higher *k*_*an*_ levels seem to produce faster resolution similar to increasing the transition rate *k*_*m*1*m*2_. This is unsurprising given the dependency of the second term of Eq 1 on *k*_*m*1*m*2_, *k*_*anm*1_, and *AN*, which tracks the size of the apoptotic neutrophil population. Yet the magnitude of the effects of modulating *k*_*an*_ versus acting on transition directly via *k*_*m*1*m*2_ are predicted to diverge for lower values, with the former providing more dramatic changes.

**Fig 10:**
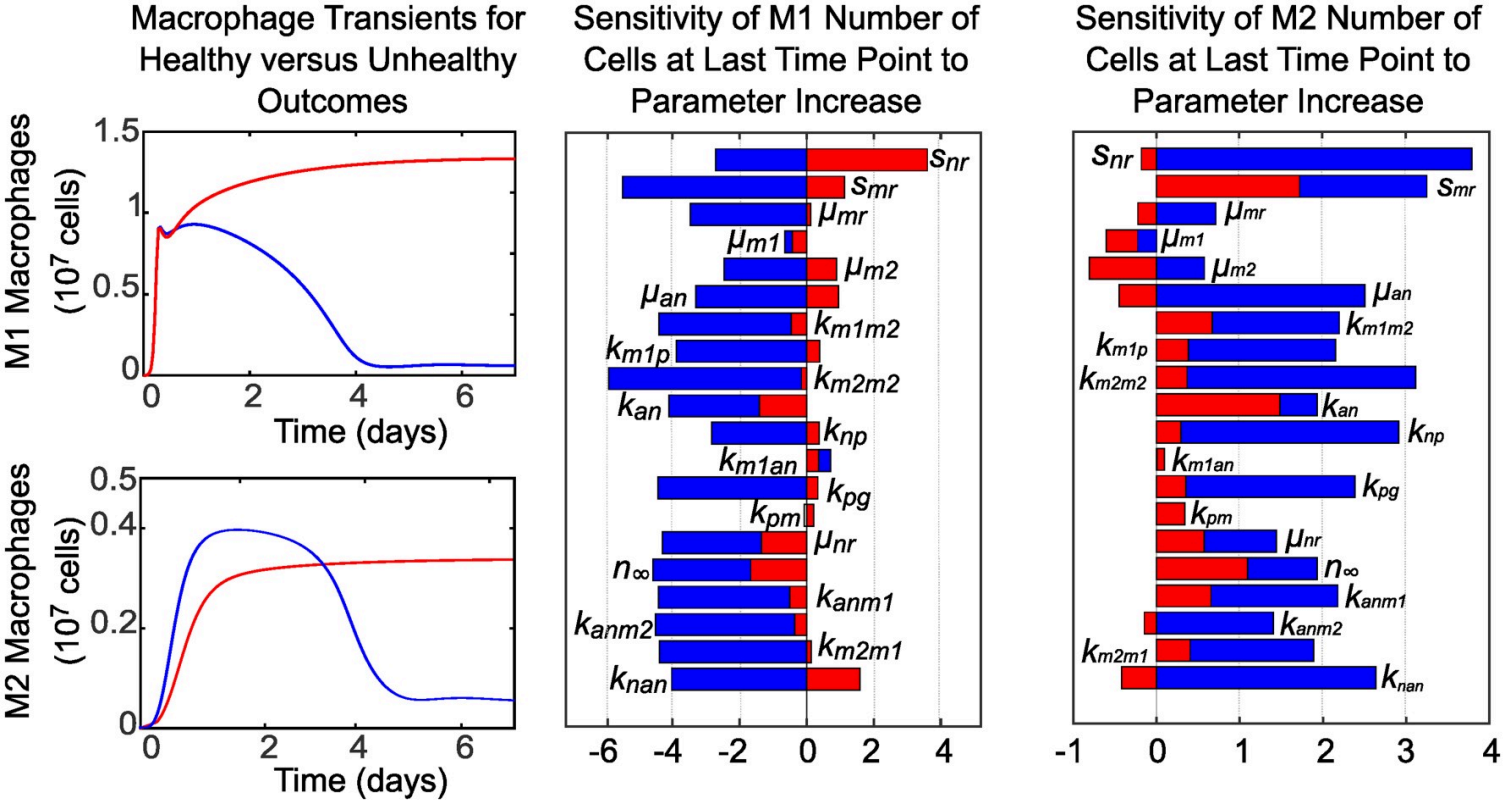
Sensitivity of M1 and M2 characteristics to parameter variations in the case of delayed neutrophil apoptosis (unhealthy response) versus a healthy response. Predictions and sensitivities for a healthy response are plotted in blue, while predictions and sensitivities for an unhealthy response are plotted in red. A healthy M1 and M2 response that resolves, with all parameters at baseline values given in Tables 1 and 4 (including *k*_*an*_ = 7.108), is plotted versus an unhealthy, sustained M1 and M2 response resulting from reducing the value of parameter *k*_*an*_ to 5.56 while holding all other parameters constant. The bar charts compare the associated sensitivity of M1 and M2 characteristics to parameter variations in the healthy case versus the unhealthy case. Insensitive parameters, which have zero sensitivity for all characteristics, are not shown.

To explore points of intervention in the case of delayed neutrophil apoptosis, we set *k*_*an*_ = 5.56. This results in sustained inflammation as shown in Fig 9, and changes in sensitivity to parameters across this bistability is also shown in Fig 10. For example, with delayed neutrophil apoptosis (unhealthy case), the number of M1s remaining at day 7 becomes strongly positively associated with parameter *s*_*nr*_ (influx rate of resting neutrophils) and the number of M2s remaining at day 7 becomes negatively associated with increased *μ*_*m*2_.

**Fig 11:**
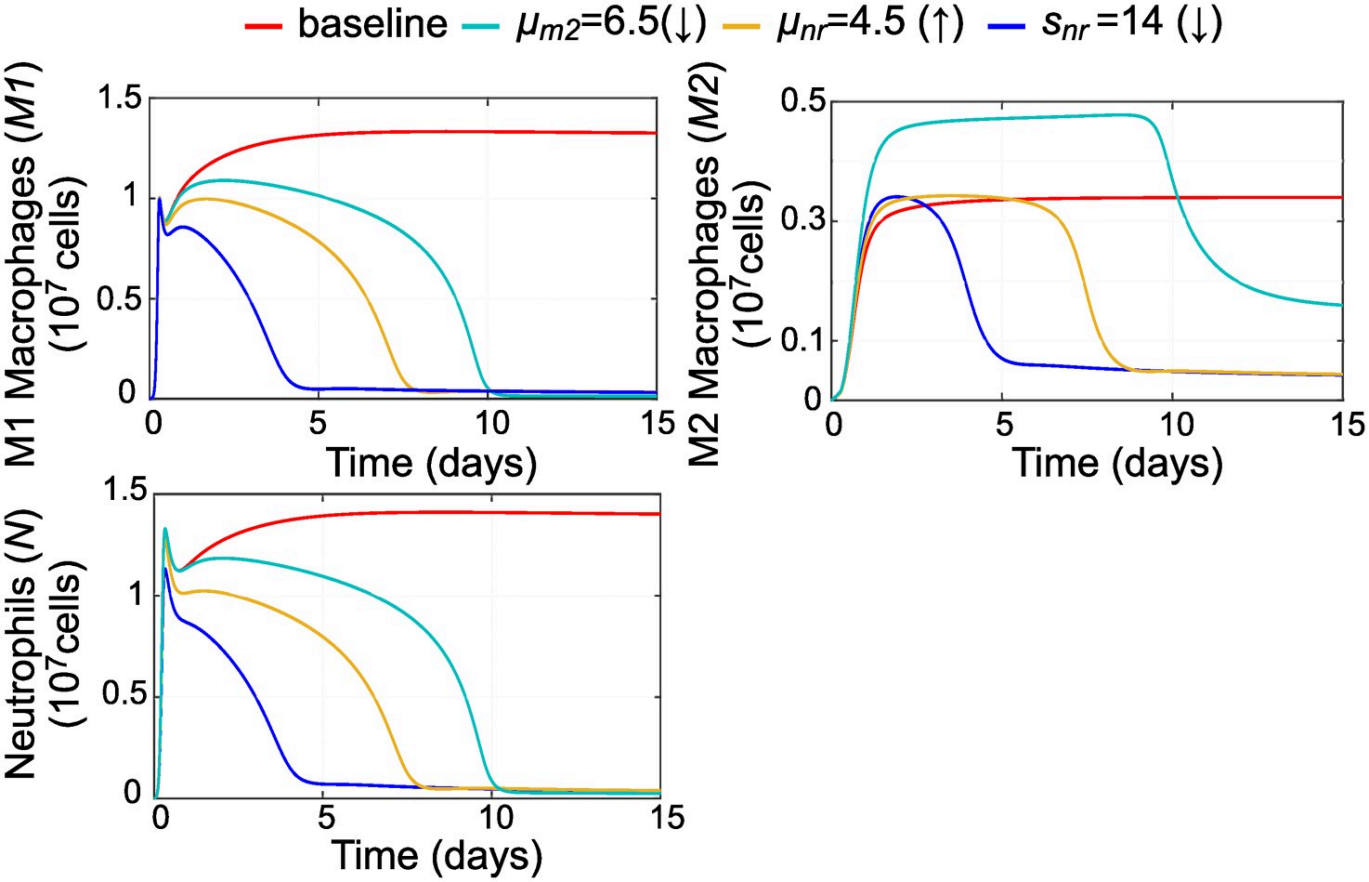
Parameter variations that resolve inflammation in the case of delayed neutrophil apoptosis. Reducing the value of parameter *k*_*an*_ from baseline while holding all other parameters constant leads to sustained inflammation. We resolved inflammation in this case by varying each of three parameters separately: *μ*_*m*2_, *u*_*nr*_, or *s*_*nr*_. All immune cells return to low levels if resting neutrophil influx or decay is modulated, while a population of M2 macrophages persists if M2s are directly targeted to resolve the inflammation.

By changing these as shown in Fig 11 we are able to resolve inflammation in spite of impaired neutrophil apoptosis. Modulating resting neutrophils by either reducing influx (simulated by lowering the value of *s*_*nr*_) or increasing decay or efflux (simulated by increasing the value of *u*_*nr*_) returns all immune cell populations to homeostasis. However, reducing decay or efflux of M2s (by lowering the value of *μ*_*m*2_) led to a resolution of inflammation but a sustained M2 population that could potentially be problematic.

**Fig 12:**
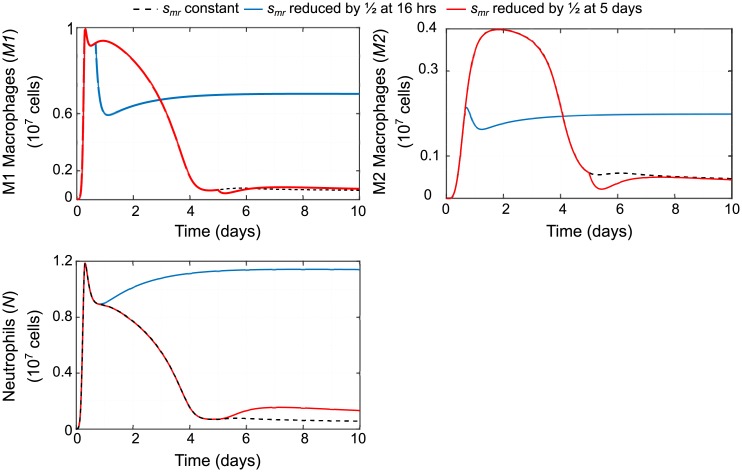
Predicted effects of reducing source of monocytes *s*_*mr*_. The effects of the baseline case of a constant influx of resting monocytes (that will differentiate into macrophages) is compared to the effects of reducing influx of monocytes at an early timepoint (16 hours) versus a late timepoint (5 days). Early intervention leads to sustained inflammation while late intervention leads to an increase in neutrophils.

Finally, we simulated reducing availability of monocytes for recruitment by reducing monocyte source parameter, *s*_*mr*_, by 1/2 at early versus late time points (16 hours or 5 days) to compare effects as shown in Fig 12. Resulting predictions support what has been demonstrated experimentally: that intervening at early timepoints to block or reduce monocyte recruitment and their subsequent differentiation to inflammatory macrophages can actually impair resolution of inflammation [60, 66, 67].

## Discussion

Modulating macrophage subpopulations has been proposed as a strategy to resolve inflammation [60–62, 68], but the mechanisms driving macrophage phenotypic switch are not well understood. In this work we have developed a model that includes macrophage polarization during inflammation. To our knowledge, it is the first model of its kind to be fit to *in vivo* experimental data. Our model allows some insight into key drivers of macrophage population shift over the time course of inflammation and allows us to predict the effects of therapies targeting macrophages.

The experimental data used to fit this mathematical model was obtained from the widely studied peritonitis model of inflammation. In addition to recapitulating the influx and egress of inflammatory cells in response to stimulus-induced inflammation, this model is also extensively used to assess the involvement of endogenous processes in mounting as well resolving the inflammatory processes. In recent studies, the pro-inflammatory role of human proteinase 3 (PR3) during acute inflammatory responses by modulating neutrophil accumulation and the underlying mechanisms were almost entirely determined using a zymosan-induced peritonitis model [69]. Extending the investigations into endogenously produced pro-resolving lipid mediators, Ramon et al. not only identified PCTR1, a member of the protectin family as a potent monocyte/macrophage agonist but also established the therapeutic potential of PCTR1 supplementation in resolving inflammation using microbial-induced peritonitis in mice [70]. Similarly, Juhas et al. confirmed the ability of RX-207 to reduce neutrophil migration using thioglycollate-induced peritonitis [71]. These examples not only underscore the importance of developing a mathematical model based on experimental data from mouse peritonitis, but also provide the rationale and future application of such a model for evaluating and predicting outcomes to be validated by subsequent experimentation.

The process of parameter selection is fully elucidated (see Results). Parameter estimation was carefully conducted such that unidentifiable parameters were fixed and the confounding effects of parameter interactions were reduced in order to obtain an identifiable subset of parameters of interest for estimation. We also stipulate that other, equally viable, identifiable subsets could have been estimated (see Table 3) and that estimated parameters may be conditional on parameters that were previously fixed. It is important to acknowledge that parameters chosen for estimation will depend on the experimental context and available measurements.

It is hypothesized that efferocytosis of apoptotic cells is an important determinant of macrophage phenotype [1, 3, 40–42], and our sensitivity analysis supports the dependence of macrophage behavior on neutrophils. Our analysis indicates that timing and magnitude of the M2 response in particular is closely related to neutrophil dynamics.

We simulated several treatment scenarios targeting macrophages both directly and indirectly. We compared the effects of targeting macrophage transition rate directly (in the model via parameter *k*_*m*1*m*2_) versus varying neutrophil apoptosis rate, *k*_*an*_, in order to increase the population of apoptotic cells available for macrophage efferocytosis. A shorter time course of both M1 and M2 response is predicted in both cases; whether this indicates fast resolution or introduces the possibility of an insufficient M2 population given a sustained pathogen insult or injury requires further examination.

Our model predicts that timing may be critical in blocking or reducing availability of monocytes in order to reduce the inflammatory M1 response, as has been proposed, and that this could lead to chronic inflammation. These effects have been observed in an experimental setting as well [60, 66, 67].

Since pro- and anti-inflammatory mediators could not be measured experimentally, we instead used cellular feedback loops to describe their contribution to inflammatory processes. The future addition of parameters such as local production/levels of pro- or anti-inflammatory mediators that likely influence the function of infiltrated immune cells will further fine-tune this model. It is noteworthy that using the mouse model of peritonitis, Dequine et al. demonstrated that local TNFR1 signaling modulated neutrophils for increased cytokine production with implications on neutrophil recruitment and egress [72]. Further experimentation is also likely to allow a larger identifiable subset of parameters, especially if cytokines associated with the various cell types are explicitly measured, giving a stronger connection between available data and feedback loop components in the model.

In future work, this peritonitis model will be extended to the case of early atherosclerosis. In addition to the routinely monitored changes in serum lipid profiles, changes in monocytosis as well as increased circulation of pro-inflammatory mediators are also causally related to atherogenesis and chronic unresolved inflammation is recognized as an underlying cause of multiple metabolic diseases. It is noteworthy that Angsana et al. reported a positive correlation between delayed clearance of macrophages from the peritoneal cavity and atherosclerotic plaque burden [73] and Feige et al. showed that a small molecule lecinoxoid (VB-201) which reduced monocyte migration in a peritonitis model, also reduced atheroma development [74]. These studies underscore the predictive value of computational models based on cellular influx/egress from the peritoneal cavity.

Chronic inflammatory diseases in general require timely peaks and ebbs in immune cell response in order for homeostastis to be restored; particularly in macrophages, which include subpopulations that either contribute to or resolve inflammation. In the case of atherosclerosis, this phenotype switching is believed to be critical to a balanced response to hyperlipidemia. Our extended model will be able to provide hypothesis testing for points of intervention in atherosclerosis that target macrophage phenotype. Jacinto et al. have recently demonstrated the importance of extra-arterial contributors such as functionality of monocytes in aggravation of atherosclerosis under normocholesterolemic conditions emphasizing the need for the inclusion of such measures into predictive models [75]. This work could also be extended to other disease systems that feature chronic inflammation, and the modeling of variables pathogen and nutrient broth could be replaced by an inflammatory stimulus input function *f*(*t*) that is more general and applicable to pathogen insult or injury.

In conclusion, data presented herein describes the development of a computational model of the sequential influx of immune cells in response to an external trigger and fitting this model to experimental data obtained from a well-established *in vivo* model of inflammatory response namely peritonitis. Fine tuning this model with inclusion of other systemic parameters related to inflammation will permit the future application to chronic inflammatory diseases with dysfunctional resolution of inflammation.

## Supporting information

S1 TableExperimental data from mouse model of peritonitis.At each time point, cells were harvested from a sample of *n* mice. Average cell counts for neutrophils ($\bar{N}$), M1 macrophages ($\bar{M1}$), and M2 macrophages ($\bar{M2}$) are given in units of (10^7^) cells. Standard error of the mean for each cell type *x* is calculated as $\sigma_{\bar{x}}=\sigma_{x}/\sqrt{n}$.(PDF)

S1 Figures Model predictions versus observations and profile likelihood plots. Model predictions versus observations are plotted for M1 macrophages (*M*1), M2 macrophages (*M*2), and neutrophils (*N*). Data points are labeled with time (in days).
(PDF)

S1 File MATLAB model definition file for PottersWheel toolbox. Installation of the Potterswheel toolbox requires extensive setup as detailed on the software installation webpage (https://potterswheel.de/Pages/installation.php) which also contains troubleshooting steps. The authors did not contribute to the development of the software.
(M) Click here for additional data file.

S2 File Excel data file for PottersWheel toolbox. (XLS)

S3 File SBML model file. This file can be translated to a readable format with online tool SbmlViewer at http://sv.insysbio.com/ and can be loaded into any SBML-compatible software. A list of software can be found at http://sbml.org.
(XML)

S4 File MATLAB main model file. This MATLAB script (solve_sys_ODEs.m) solves the system of ODEs presented in this paper and plots model predictions versus data.
(M) Click here for additional data file.

S5 File MATLAB model ODEs. This MATLAB function encodes the system of ODEs presented in this paper and is called by the main model file.
(M) Click here for additional data file.

S6 File MATLAB data file. This MATLAB file contains the data presented in this paper and is called by the main model file.
(M) Click here for additional data file.
