# Supplementary material for: Identifying important parameters in the inflammatory process with a mathematical model of immune cell influx and macrophage polarization
Source: PLoS Comput Biol. 2019 Jul 31;15(7):e1007172. doi: 10.1371/journal.pcbi.1007172 (PMC6690555; doi:10.1371/journal.pcbi.1007172)
Supplement: S1 Table — At each time point, cells were harvested from a sample of n mice. Average cell counts for neutrophils (N¯), M1 macrophages (M1¯), and M2 macrophages (M2¯) are given in units of (107) cells. Standard error of the mean for each cell type x is calculated as σx¯=σx/n. (PDF) [file pcbi.1007172.s001.pdf]

S1 Table. Experimental data from mouse model of peritonitis. At each time point, cells were harvested from a sample of n mice. Average cell counts for neutrophils ($\overline{N}$), M1 macrophages ($\overline{M1}$), and M2 macrophages ($\overline{M2}$) are given in units of (10^7) cells. Standard error of the mean for each cell type x is calculated as $\sigma_{\overline{x}} = \sigma_x/\sqrt{n}$.

**Hours**	n	$\overline{N}$	$\overline{M1}$	$\overline{M2}$	σ_N	$\sigma_{\overline{N}}$	σ_{M1}	$\sigma_{\overline{M1}}$	σ_{M2}	$\sigma_{\overline{M2}}$
16	8	0.868	0.835	0.214	0.316	0.112	0.311	0.110	0.063	0.022
20	8	0.798	0.834	0.266	0.302	0.107	0.322	0.114	0.136	0.048
24	8	1.034	1.106	0.380	0.363	0.128	0.388	0.137	0.145	0.051
40	5	0.653	0.816	0.349	0.378	0.169	0.499	0.223	0.244	0.109
48	8	0.800	0.890	0.457	0.350	0.124	0.396	0.140	0.245	0.087
72	4	0.600	0.625	0.409	0.425	0.213	0.396	0.180	0.106	0.053
96	8	0.185	0.083	0.174	0.109	0.039	0.033	0.012	0.112	0.040
120	8	0.076	0.035	0.081	0.055	0.019	0.022	0.008	0.067	0.024
144	8	0.146	0.0895	0.091	0.141	0.050	0.165	0.058	0.075	0.027
168	8	0.042	0.027	0.029	0.026	0.009	0.038	0.013	0.02	0.007

Table 1. Experimental data from mouse model of peritonitis.
