# Supplementary material for: Identifying important parameters in the inflammatory process with a mathematical model of immune cell influx and macrophage polarization
Source: PLoS Comput Biol. 2019 Jul 31;15(7):e1007172. doi: 10.1371/journal.pcbi.1007172 (PMC6690555; doi:10.1371/journal.pcbi.1007172)

Fig 1. Model predictions versus observations. Model predictions versus observations are plotted for M1 macrophages ($M1$), M2 macrophages ($M2$), and neutrophils (N). Data points are labeled with time (in days).

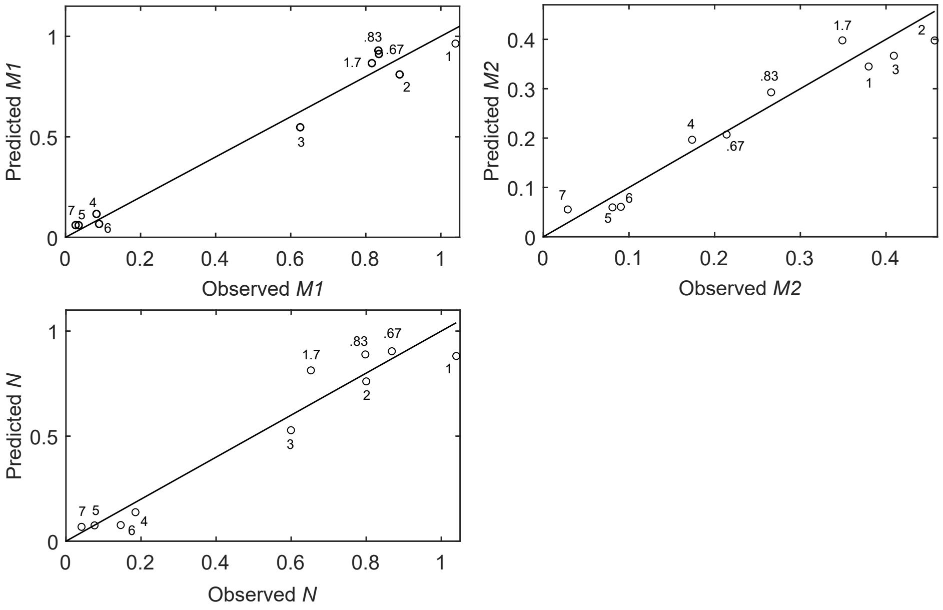

Fig 2. Profile likelihood plots. The six parameters in the identifiable subset are plotted versus the estimated parameters on a logarithmic scale. Only estimated parameters that change significantly are plotted. These changes can indicate dependencies between parameters.

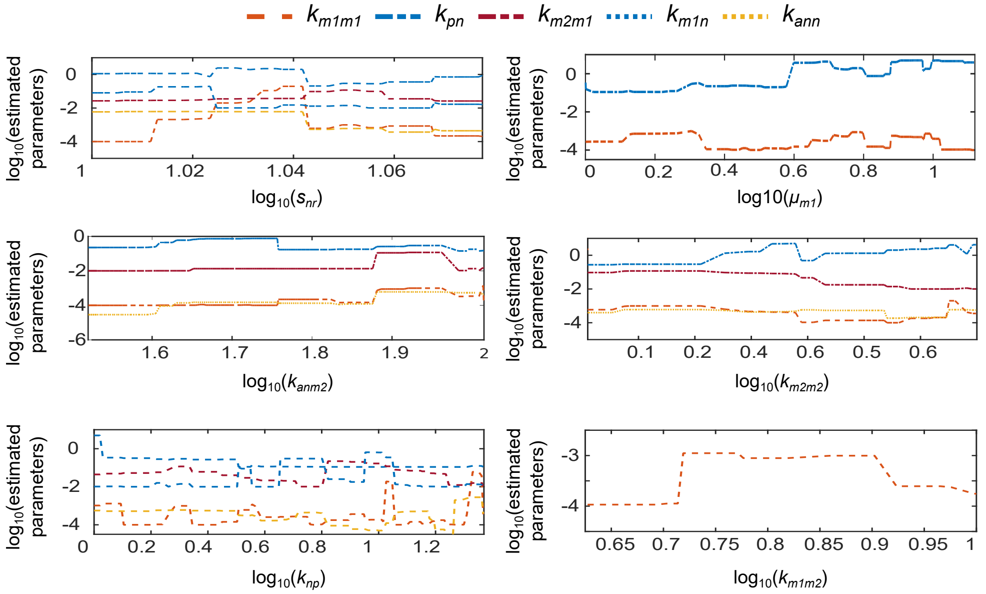

Supplement: S1 Figures — Model predictions versus observations are plotted for M1 macrophages (M1), M2 macrophages (M2), and neutrophils (N). Data points are labeled with time (in days). (PDF) [file pcbi.1007172.s002.pdf]
